# Effect of ulinastatin on post-operative blood loss and allogeneic transfusion in patients receiving cardiac surgery with cardiopulmonary bypass: a prospective randomized controlled study with 10-year follow-up

**DOI:** 10.1186/s13019-020-01144-9

**Published:** 2020-05-14

**Authors:** Peng Zhang, Hong Lv, Xia Qi, Wenjing Xiao, Qinghua Xue, Lei Zhang, Lihuan Li, Jia Shi

**Affiliations:** 1 Department of Surgery, Fuwai Hospital, National Center for Cardiovascular Diseases, Chinese Academy of Medical Sciences and Peking Union Medical College, 167 Beilishi Rd., Xicheng District, Beijing, 100037 China; 2 Department of Anaesthesiology, State Key Laboratory of Cardiovascular Disease, Fuwai Hospital, National Center for Cardiovascular Diseases, Chinese Academy of Medical Sciences and Peking Union Medical College, 167 Beilishi Rd., Xicheng District, Beijing, 100037 China; 3 Department of Anaesthesiology, People’s Hospital of Ningxia Hui Autonomous Region, 148 Huaiyuanxi Rd. Xixia District, Ningxia Hui Autonomous Region, Yinchuan, 750021 China

**Keywords:** Ulinastatin, Tranexamic acid, Blood conservation, Cardiopulmonary bypass

## Abstract

**Background:**

Major bleeding and allogeneic transfusion leads to negative outcomes in patients receiving cardiac surgery with cardiopulmonary bypass (CPB). Ulinastatin, a urine trypsin inhibitor, relieves systemic inflammation and improves coagulation profiles with however sparse evidence of its effects on blood loss and allogeneic transfusion in this specific population.

**Methods:**

In this prospective randomized controlled trial, 426 consecutive patients receiving open heart surgery with CPB were randomly assigned into three groups to receive ulinastatin (group U, *n* = 142), tranexamic acid (group T, *n* = 143) or normal saline (group C, *n* = 141). The primary outcome was the total volume of post-operative bleeding and the secondary outcome included the volume and exposure of allogeneic transfusion, the incidence of stroke, post-operative myocardial infarction, renal failure, respiratory failure and all-cause mortality. A ten-year follow-up was carried on to evaluate long-term safety.

**Results:**

Compared with placebo, ulinastatin significantly reduced the volume of post-operative blood loss within 24 h (688.39 ± 393.55 ml vs 854.33 ± 434.03 ml MD − 165.95 ml, 95%CI − 262.88 ml to − 69.01 ml, *p* < 0.001) and the volume of allogeneic erythrocyte transfusion (2.57 ± 3.15 unit vs 3.73 ± 4.21 unit, MD-1.16 unit, 95%CI − 2.06 units to − 0.26 units, *p* = 0.002). The bleeding and transfusion outcomes were comparable between the ulinastatin group and the tranexamic acid group. In-hospital outcomes and 10-year follow-up showed no statistical difference in mortality and major morbidity among groups.

**Conclusions:**

Ulinastatin reduced post-operative blood loss and allogeneic erythrocyte transfusion in heart surgery with CPB. The mortality and major morbidity was comparable among the groups shown by the 10-year follow-up.

**Trial registration:**

The trial was retrospectively registered on February 2, 2010. Trial registration number: https://www.clinicaltrials.gov Identifier: NCT01060189.

## Background

Excessive bleeding and allogeneic transfusion is a major concern in cardiac surgical procedures leading to deteriorated overall outcomes [1, 2] and worsened long-term mortality [3]*.* Open heart surgery with cardiopulmonary bypass (CPB) and aortic cross-clamping produces variable systemic inflammatory reactions [4–7], which are associated with multi-organ dysfunction via the action of leucocytes, especially polymorphonuclear neutrophils (PMNs) [8]. The PMNs degrade or inhibit the activity of fibrin, fibrinogen, platelets and coagulation factors, [9–11] and lead to increased blood loss and demand for transfusion [12].

Ulinastatin is a urinary trypsin inhibitor, which is extracted and purified from fresh healthy human urine [13]. Ulinastatin decreases the release of elastase from PMNs and suppresses elastase activity [14].. An in vivo study showed that ulinastatin also stabilized lysosomal membranes and inhibited the release of lysosomal enzymes [15]. Ulinastatin ameliorated preoperative coagulopathy and normalized thromboelastography in patients with liver resection [16]. Furthermore, ulinastatin shortened activated partial thromboplastin time (aPTT) and activated coagulation time (ACT) in patients undergoing cardiopulmonary bypass [17]. Ulinastatin is a protease inhibitor, which is similar to aprotinin, therefore ulinastatin is expected to decrease post-operative bleeding. However two small-sized studies showed no improvement in blood loss and transfusion sparing in patients undergoing specific open heart surgery pretreated with ulinastatin [18, 19].

Tranexamic acid (TXA) can effectively reduce post-operative bleeding and demand for transfusion [20–22]. TXA is a lysine analogue that prevents degradation of fibrin and dissolution of clots by inhibiting the activation of plasminogen. In 2008 aprotinin was removed from the market [23], before TXA became the mainstay of anti-fibrinolytic therapy for pharmacological blood conservation in cardiac surgery, other alternatives had been exploring for. Given that ulinastatin was a protease that was similar to aprotinin, it became a hopeful candidate. However, few studies were reported on its effect on blood conservation and short- and long-term outcomes.

Therefore, the aim of the current study was to evaluate the efficacy of ulinastatin on post-operative blood loss and allogeneic transfusion in comparison with the tranexamic acid as positive control and placebo as negative control.

## Methods

### Trial design

The study was a prospective, randomized, double-blinded and controlled trial. It was sponsored by National Center for Cardiovascular Diseases and was conducted at Fuwai Hospital, Chinese Academy of Medical Sciences and Peking Union Medical College. The study was approved by the Ethical Review Board of Fuwai Hospital. (Ethical approval No. 2008–366). And written informed consent was provided by all participants.

### Study population

The inclusion criteria were patients between 18 and 79 years old undergoing elective heart surgery with cardiopulmonary bypass, including the coronary artery bypass graft, valvular repair or replacement, or repair of congenital heart deformities. The exclusion criteria included previous cardiac surgery, hematocrit level less than 33%, platelet count less than 100,000 × 10^3^/L, allergy to tranexamic acid, and being recruited in other studies.

### Randomization and blinding

The surgical procedures and peri-operative care followed the institutional routine. Aspirin and clopidogrel, if any, were discontinued at least 5 days before the operation. In patients using warfarin, it was required that prothrombin time (PT) was normal before the operation. Patients were randomly assigned into three groups for the use of ulinastatin (group U), tranexamic acid (group T) or placebo (group C). The randomization sequence was generated by computer in permuted blocks by a 1:1:1 ratio and was masked in sealed, sequentially numbered and opaque envelopes. Patient enrollment, randomization, and blinding were conducted and supervised by an independent committee. The participants, medical staff, and investigators were unaware of the treatment allocation until the end of the study.

### Primary and secondary outcomes

The primary outcome of this study was the total volume of post-operative blood loss. The secondary outcomes included stroke, post-operative myocardial infarction, renal failure, respiratory failure, in-hospital adverse outcomes and long-term morbidities and mortalities. Stroke was stated as new focal neurologic deficit lasting more than 24 h confirmed by a cerebral computed tomography scan and an attending neurologic consultant. Post-operative myocardial infarction was diagnosed by two of the following: prolonged (> 20 min) chest pain not relieved by rest or nitrates, new pathologic Q waves in more than two contiguous electrocardiograph leads, elevated enzyme levels (creatine kinase-MB > 5% of total creatinine phosphokinase or troponin T > 0.5 ng/mL), new wall motion abnormalities, or the need for revascularization. Renal failure was stated as first-time dependency on renal dialysis, an increase of post-operative creatinine of at least 2 mg/dL, or a difference of at least 0.7 mg/dL between baseline value and the maximal post-operative plasma creatinine concentration. Respiratory failure was defined as prolonged mechanical ventilation (> 48 h), the need for continuous positive airway pressure therapy, reintubation, or tracheostomy. The in-hospital adverse outcomes were evaluated and defined as seizure, sudden cardiac arrest, readmission to intensive care unit (ICU), re-operation for surgical cause, using intra-aortic balloon pulsation (IABP) or extracorporeal membrane oxygenation (ECMO) and deep sternal infection. The long-term morbidities included stroke, myocardial infarction, renal failure, respiratory failure, seizure and sudden cardiac arrest.

### Interventions

Study and placebo medication were prepared by the hospital pharmacy. Identical syringes of 50 mL labeled with the randomization number contained transparent solution, 30 mg/Kg body weight of tranexamic acid (Jie Ning®; Changchun Tiancheng Pharmaceutical Co., Changchun, China), 1,000,000 U ulinastatin (Tian Pu Luo An®; Guangdong Tianpu Biochemistry Pharmaceutical Co., Guangzhou, China) or normal saline. There were two syringes prepared for each patient labeled ‘#1’ and ‘#2’. In the tranexamic acid group, both the syringes contained 15 mg/kg tranexamic acid to fulfill a total dosage of 30 mg/kg. In the ulinastatin group, the syringe #1 contained 1,000,000 U ulinastatin and the syringe #2 contained normal saline. In the control group, both the syringes contained normal saline. The syringe #1 and #2 were pumped intravenously after anaesthetic induction and after the administration of protamine respectively.

### Post-operative blood loss

Postoperative blood loss was assessed via chest drain tubes every 8 h for the first 24 h after admittance to the ICU, and then was assessed every day beyond the first 24 h until the chest drain tubes were withdrawn. Post-operative blood loss was defined as the total volume of drainage from the end of the operation until the removal of the chest tubes. Chest tube drainage more than 300 ml within the first post-operative hour, more than 5 ml/kg per hour consecutively for 3 h, or any signs of pericardial tamponade justified surgical re-exploration to control bleeding.

### Transfusion criteria

The criteria for the transfusion of packed red blood cells (RBC) were:

- (1) bleeding caused hemodynamic instability or
- (2) hemoglobin concentrations below 8.0 g/dL in the early postoperative period

In all other situations the decision to transfuse was left to the discretion of the treating physician. The criteria for transfusion of platelet concentrates and fresh frozen plasma were:

- (1) excessive bleeding and a platelet count < 50000/L or
- (2) PT and/or aPTT of > 1.5 times the upper limit of normal (after heparin reversal), respectively

Additional protamine was administered in cases of prolonged ACT (the preoperatively measured ACT served as reference).

### Follow-up

All patients were followed up for 10 years via reviewing outpatient records and questionnaires by mail/telephone 30 days post-operatively and annually.

### Statistical analysis

The sample size was calculated based on the volume of post-operative bleeding using one-way ANOVA at an alpha level of 0.05 and effect size of 0.2 with 95% power. Assuming a dropout rate of 10%, the estimated total sample size was 426 patients (142 patients for each group). For continuous variables, normal distribution assumption was assessed. Equal variance assumption was assessed. The differences of these characteristics between groups were performed using independent two-sample t-tests and one-way ANOVA. Mean difference (MD) and its 95% confidence interval (CI) was calculated. Categorical variables were summarized using frequency and percentage and compared using Chi-square test or Fisher’s exact test. The estimated effect size and its precision were presented by the absolute risk difference (RD) and relative risk (RR) with their associated 95% CIs. The Mantel- Haenszel method was applied in the calculation of RR. Survival analysis was performed using the Kaplan-Meier method and log-rank test. All the analyses were performed using SPSS (Version 18.0, SPSS Inc.) software. All tests were two-sided, and a probability value less than 0.05 was considered to be statistically significant. The authors had full access to the data and take responsibility for its integrity.

## Results

### Baseline characteristics and peri-operative data of the study subjects

**Fig. 1:**
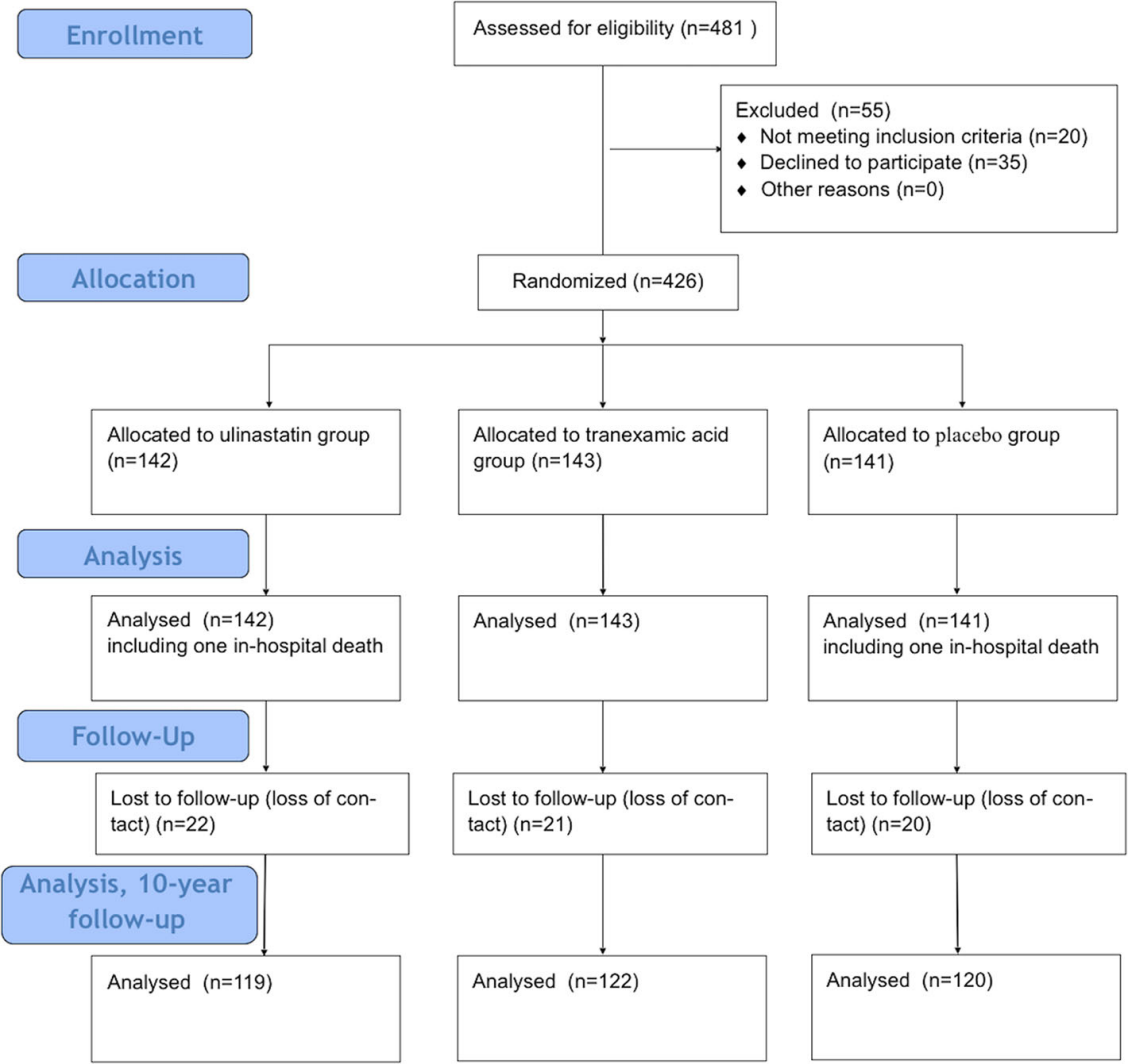
CONSROT flow chart

From April 2008 to Dec 2008, a total of 481 patients were eligible for access in the present study. Of 481 patients, 55 patients were excluded. Twenty patients didn’t meet the inclusion criteria and 35 patients refused to participate. (Fig. 1) The remained 426 patients were randomized to receiving ulinastatin (Group U, *n* = 142), tranexamic acid (Group T, *n* = 143), or placebo (Group C, *n* = 141). The baseline characteristics of each group are shown in Table 1. (Table 1)There was no statistical significant difference in demographic characteristics, main diagnoses and preoperative comorbidity among the groups. Types of operations included on-pump coronary artery bypass graft (CABG), valvular procedures and congenital deformity repairs. There was no significant difference among groups in terms of the constitution of surgical procedures, CPB time and aortic cross-clamping time as seen in Table 2. (Table 2)No significant difference was found in mechanical ventilation time, chest tube removal time, ICU stay and hospital length of stay.

**Table 1:** Baseline characteristics of all patients

	Ulinastatin group (*n* = 142)	Tranexamic acid group (*n* = 143)	Placebo group (*n* = 141)	p
Baseline demographics				
Male n,(%)	63 (44.37%)	66 (46.15%)	71 (50.35%)	0.585
Age (yrs)	49.0 ± 14.3	48.6 ± 12.7	50.3 ± 12.4	0.562
Weight (kg)	61.7 ± 11.7	61.4 ± 12.0	61.4 ± 12.7	0.907
Body mass index	23.1 ± 3.6	22.9 ± 3.4	22.8 ± 3.7	0.686
Diagnosis				0.631
Coronary heart disease, n(%)	17 (11.97%)	13 (9.09%)	21 (14.89%)	
Valvular heart disease, n(%)				
Mitral valve lesion, n(%)	70 (49.30%)	66 (46.15%)	63 (44.68%)	
Aortic valve lesion, n(%)	24 (16.90%)	23 (16.08%)	19 (13.48%)	
Combined lesion, n(%)	21 (14.79%)	19 (13.29%)	19 (13.48%)	
Congenital heart disease, n(%)				
Atrial septal defect, n(%)	1 (0.70%)	4 (2.80%)	6 (4.26%)	
Ventricular septal defect, n(%)	4 (2.82%)	6 (4.20%)	5 (3.55%)	
Other, n(%)	5 (3.52%)	12 (8.39%)	8 (5.67%)	
Clinical history (%)				
Hypertension	31 (21.83%)	33 (23.08%0	37 (26.24%)	0.668
Diabetes	5 (3.52%)	4 (2.80%)	6 (4.26%)	0.801
Stroke	7 (4.93%)	3 (2.10%)	2 (1.42%)	0.166
Chronic pulmonary disease	10 (7.04%)	19 (13.29%)	15 (10.64%)	0.221
Liver dysfunction	0 (0%)	0 (0%)	0 (0%)	–
Renal dysfunction	0 (0%)	1 (0.70%)	2 (1.42%)	0.361
NYHA class (%)				0.610
I	10 (7.04%)	18 (12.59%)	18 (12.77%)	
II	81 (57.04%)	75 (52.45%)	78 (55.32%)	
III	41 (28.87%)	36 (25.17%)	35 (24.82%)	
IV	10 (7.04%)	14 (9.79%)	10 (7.09%)	
Euroscore II	2.41 ± 1.87	2.63 ± 1.73	2.50 ± 1.75	0.549

**Table 2:** Peri-operative data

	Ulinastatin group (*n* = 142)	Tranexamic acid group (*n* = 143)	Placebo group (*n* = 141)	P
Surgical procedures				0.733
On-pump coronary artery bypass grafting, n (%)	16 (11.27%)	13 (9.09%)	21 (14.89%)	
Mitral valvuloplasty/replacement, n (%)	66 (46.48%)	65 (45.45%)	60 (42.55%)	
Aortic valve replacement, n (%)	23 (16.20%)	21 (14.69%)	19 (13.48%)	
Mitral and aortic valve replacement, n (%)	17 (11.97%)	15 (10.49%)	15 (10.64%)	
Coronary bypass and valvular surgery, n (%)	10 (7.04%)	7 (4.90%)	7 (4.96%)	
Repair of atrial septal defect, n (%)	1 (0.70%)	4 (2.80%)	6 (4.26%)	
Repair of ventricular septal defect, n (%)	4 (2.82%)	6 (4.20%)	5 (3.55%)	
Other	5 (3.52%)	12 (8.39%)	8 (5.67%)	
Operative data				
Total dose of heparin (IU/kg)	28,331.69 ± 6695.96	28,729.51 ± 6612.39	28,653.19 ± 6738.07	0.924
Total dose of protamine (mg)	393.28 ± 111.35	408.17 ± 117.55	407.87 ± 111.6	0.415
Heparin neutralization ratio	1.41 ± 0.34	1.45 ± 0.38	1.46 ± 0.39	0.510
Aortic cross-clamp time (min)	75.23 ± 34.8	68.77 ± 34.56	72.32 ± 38.09	0.186
CPB time (min)	102.87 ± 42.74	95.60 ± 42.07	98.69 ± 47.76	0.205
Chest closure time (min)	66.71 ± 30.44	64.04 ± 24.8	67.04 ± 30.93	0.888
Operation time (min)	216.8 ± 64.03	206.46 ± 62.25	205.28 ± 65.36	0.156
Inotropic support, n (%)	141 (99.3%)	140 (97.9%)	138 (97.87%)	0.559
Operative mortality	0	0	0	–
Postoperative time course				
Mechanical ventilation (hrs)	15.0 ± 7.66	14.92 ± 10.58	16.95 ± 20.85	0.609
ICU stay (hrs)	38.94 ± 26.91	38.06 ± 29.94	42.23 ± 35.3	0.741
Chest tube removal (hrs)	50.39 ± 25.68	49.92 ± 18.29	48.01 ± 23.54	0.649
Hospital length of stay (days)	8.24 ± 3.19	8.05 ± 2.46	8.51 ± 2.79	0.338

### Bleeding and transfusion

There were significant differences among three groups regarding to the blood loss within 24 h post-operatively, the total blood loss, major bleeding, reoperations, and the amount of RBC and plasma transfusion. (Table 3) In post hoc analyses (Table 3), postoperative blood loss within 24 h (404.87 ± 253.58 ml vs. 527.73 ± 300.4 ml, MD − 122.86 ml, 95% CI − 195.87 ml to − 49.86 ml, *p* < 0.001, for the first 8 h; 183.94 ± 151.83 ml vs. 205.57 ± 129.57 ml, MD − 21.63 ml, 95% CI − 55.49 ml to 12.22 ml, *p* = 0.016, for the second 8 h; 99.58 ± 94.75 ml vs. 121.03 ± 101.62 ml, MD − 21.45 ml, 95% CI − 44.58 ml to 1.68 ml, *p* = 0.029, for the third 8 h; 688.39 ± 393.55 ml vs 854.33 ± 434.03 ml, MD − 165.95 ml, 95% CI − 262.88 ml to − 69.01 ml, *p* < 0.001, for the wholly first 24 h) and total blood loss (801.7 ± 460.14 ml vs. 1016.67 ± 529.08, MD − 214.98 ml, 95% CI − 338.60 ml to − 91.36 ml, *p* < 0.001) were significantly reduced in patients receiving ulinastatin compared with placebo. The major bleeding was comparable between group U and group C. It was a trend that there were fewer reoperations in group U than in group C (0.70% vs. 4.26%; RD -0.0355, 95% CI − 0.0716 to 0.0005; RR, 0.166; 95% CI 0.0202 to 1.36; *p* = 0.055).

**Table 3:** Bleeding and transfusion outcomes

	Ulinastatin group (*n* = 142)		Tranexamic acid group (*n* = 143)			Placebo group (*n* = 141)			P
Bleeding									
Blood loss within 8 h postoperatively (ml)	404.87 ± 253.58		380.56 ± 274.3			527.73 ± 300.4			< 0.001
Blood loss 9–16 h postoperatively (ml)	183.94 ± 151.83		165.28 ± 98.02			205.57 ± 129.57			0.004
Blood loss 17–24 h postoperatively (ml)	99.58 ± 94.75		91.08 ± 61.64			121.03 ± 101.62			0.034
Blood loss within 24 h postoperatively (ml)	688.39 ± 393.55		636.92 ± 368.87			854.33 ± 434.03			< 0.001
Blood loss beyond 25 h postoperatively (ml)	113.31 ± 108.77		111.64 ± 97.73			162.34 ± 176.69			0.096
Blood loss totality postoperatively (ml)	801.7 ± 460.14		748.57 ± 409.53			1016.67 ± 529.08			< 0.001
Major bleeding, n (%)	52 (36.62%)		42 (29.37%)			66 (46.81%)			0.010
Reoperation, n (%)	1 (0.7%)		0 (0%)			6 (4.26%)			0.011
Allogenic transfusion									
Red blood cells (unit)	2.57 ± 3.15			2.15 ± 2.7		3.73 ± 4.21			0.002
Plasma (unit)	279.61 ± 439.44			172.03 ± 298.92		382.98 ± 530.49			0.002
Platelets (unit)	0.01 ± 0.12			0.03 ± 0.2		0.06 ± 0.26			0.160
Patients exposed to allogenic blood products									
Red blood cells, n (%)	83 (58.45%)			79 (55.24%)			98 (69.50%)		0.036
Plasma, n (%)	61 (42.96%)			49 (34.27%)			71 (50.35%)		0.023
Platelets, n (%)	2 (1.41%)			3 (2.10%)			7 (4.96%)		0.159
Any, n (%)	97 (68.31%)			87 (60.84%)			104 (73.76%)		0.065
	Ulinastatin vs. Tranexamic acid			Ulinastatin vs. Placebo			Tranexamic acid vs. Placebo		
	RD (95%CI) or MD (95%CI)	RR (95%CI)	P.	RD (95%CI) or MD (95%CI)	RR (95%CI)	P	RD (95%CI) or MD (95%CI)	RR (95%CI)	P
Bleeding									
Blood loss within 8 h postoperatively (ml)	24.31 (−37.30,85.91)	–	0.148	−122.86 (−195.87,-49.86)	–	< 0.001	− 147.17 (− 220.05,-74.30)	–	< 0.001
Blood loss 9–16 h postoperatively (ml)	18.66 (− 11.11,48.44)	–	0.507	−21.63 (−55.49,12.22)	–	0.016	−40.29 (−74.09,-6.50)	–	0.001
Blood loss 17–24 h postoperatively (ml)	8.49 (−10.13,27.12)	–	0.913	−21.45 (−44.58,1.68)	–	0.029	−29.94 (−53.03,-6.85)	–	0.020
Blood loss within 24 h postoperatively (ml)	51.46 (−37.47,140.40)		0.257	−165.95 (− 262.88,-69.01)		< 0.001	− 217.41 (− 311.45,-123.37)		< 0.001
Blood loss beyond 25 h postoperatively (ml)	1.66 (−22.44,25.77)	–	0.951	−49.03 (−83.92,-14.15)	–	0.060	−50.70 (−85.52,-15.87)	–	0.062
Blood loss totality postoperatively (ml)	53.13 (−48.42,154.68)	–	0.463	− 214.98 (−338.60,-91.36)	–	< 0.001	− 268.11 (− 391.51,-144.70)	–	< 0.001
Major bleeding (person)	0.0725 (− 0.0364,0.181)	1.25 (0.893,1.74)	0.193	− 0.102 (− 0.216,0.0124)	0.782 (0.592,1.03)	0.082	− 0.174 (− 0.286,-0.0632)	0.628 (0.460,0.855)	0.002
Reoperation (person)	0.0070 (− 0.0067,0.0208)	3.02 (0.124,73.5)	0.315	−0.0355 (− 0.0716,0.0005)	0.166 (0.0202,1.36)	0.055	− 0.0426 (− 0.0759,-0.0092)	0.0759 (0.0043,1.33)	0.012
Allogenic transfusion									
Red blood cells (unit)	0.42 (−0.26,1.10)	–	0.330	−1.16 (− 2.06,-0.26)	–	0.016	−1.58 (− 2.48,-0.69)	–	0.001
Plasma (unit)	107.58 (20.00,195.16)	–	0.052	−103.37 (− 217.62,10.88)	–	0.113	− 210.95 (− 325.00,-96.90)	–	0.001
Platelets (unit)	− 0.01(− 0.05,0.02)	–	0.656	−0.04(− 0.10,0.011)	–	0.088	−0.03(− 0.08,0.02)	–	0.195
Patients exposed to allogenic blood products									
Red blood cells (person)	0.0321 (−0.0829,0.147)	1.058 (0.864,1.30)	0.585	−0.111 (− 0.222,0.0006)	0.841 (0.705,1.00)	0.053	−0.143 (− 0.254,-0.031)	0.795 (0.662,0.955)	0.013
Plasma (person)	0.0869 (−0.0257,0.200)	1.254 (0.933,1.69)	0.132	−0.0740 (− 0.190,0.0420)	0.853 (0.664,1.10)	0.212	−0.161 (− 0.274,-0.048)	0.681 (0.514,0.900)	0.006
Platelets (person)	−0.0069 (− 0.0373,0.0236)	0.671 (0.114,3.96)	0.658	−0.0356 (− 0.0763,0.0052)	0.284 (0.0600,1.34)	0.088	− 0.0287 (− 0.0715,0.0142)	0.423 (0.112,1.60)	0.190
Any (person)	0.0747 (−0.0360,0.185)	1.12 (0.945,1.34)	0.187	−0.0545 (− 0.160,0.0510)	0.926 (0.798,1.08)	0.312	−0.129 (− 0.237,-0.0212)	0.825 (0.700,0.972)	0.020

Patients in group U had less allogeneic erythrocyte transfusion compared to patients in group C (2.57 ± 3.15 units vs. 3.73 ± 4.21 units; MD -1.16; 95% CI − 2.06 to − 0.265; *p* = 0.016). Ulinastatin tended to reduce exposure to RBC in contrast to placebo (58.45% vs. 69.50%; RD -0.111, 95% CI − 0.222 to 0.0006; RR 0.841, 95% CI 0.705 to 1.00; *p* = 0.053). The volume and the exposure of plasma and platelet transfusion was similar between group U and group C. Between group U and group T, there was no significant difference in all indicators (blood loss, major bleeding, re-operation, allogenic transfusion and exposure to transfusion). (Table 3).

### Mortality and morbidity in-hospital

There was one in-hospital death in group U and in group C, respectively, and no in-hospital death in group T (0.70, 0.71%, 0, respectively; *p* = 0.602). There were significantly fewer patients who had respiratory failure post-operatively in group U (0.70, 4.20 and 6.38% in group U, group T and group C, respectively; *p* = 0.040). However, no significant difference was found among groups in other major morbidities, including stroke, postoperative myocardial infarction and renal failure. In all groups, duration of intensive care unit stay and hospital stay were similar. And no significant difference in adverse outcomes was found. (Table 4)

**Table 4:** In-hospital morbidity and mortality

	Ulinastatin group (*n* = 142)		Tranexamic acid group (*n* = 143)		Placebo group (*n* = 141)		P
	n	%	n	%	n	%	
Mortality in-hospital	1	0.70%	0	0.00%	1	0.71%	0.602
Morbidity in-hospital							
Stroke	0	0.00%	2	1.40%	1	0.71%	0.369
Postoperative MI	0	0.00%	1	0.70%	2	1.42%	0.361
Renal failure	0	0.00%	2	1.40%	4	2.84%	0.129
Respiratory failure	1	0.70%	6	4.20%	9	6.38%	0.040
Adverse outcomes in-hospital							
Seizure	0	0.00%	2	1.40%	1	0.71%	0.369
Sudden cardiac arrest	0	0.00%	1	0.70%	2	1.42%	0.361
Readmission to ICU	2	1.41%	1	0.70%	3	2.13%	0.594
Reoperation for surgical cause	2	1.41%	1	0.70%	1	0.71%	0.777
IABP	1	0.70%	2	1.40%	3	2.13%	0.597
ECMO	0	0.00%	0	0.00%	0	0.00%	–
Deep sternal infection	1	0.70%	0	0.00%	1	0.71%	0.602

### Follow-up

**Fig. 2:**
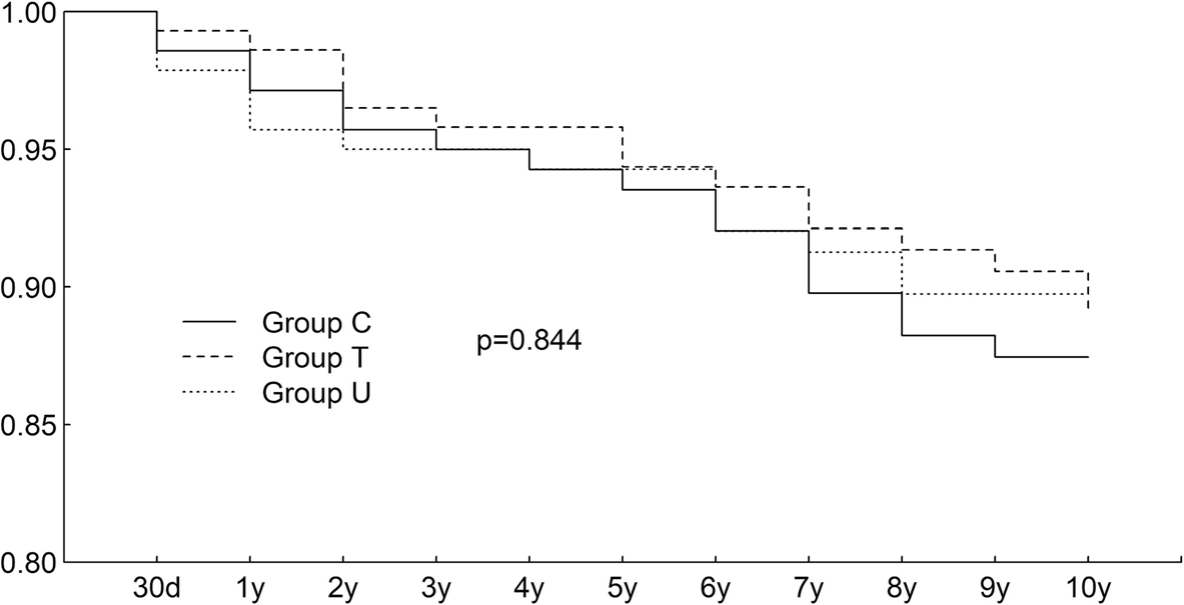
Long-term survival (10-year follow-up). There was no significant difference among three groups in long-term survival. (log-rank test, *p* = 0.844). (Group T = tranexamic acid group; Group U = ulinastatin group; Group C = placebo group)

All patients were followed up for 10 years. There was no significant difference in long-term survival among three groups. (Fig. 2) In further analyses, no significant difference in survival was found between each two groups. There was no difference in major morbidities between groups.

## Discussion

To our knowledge, for the first time the current study demonstrated that ulinastatin effectively reduced the blood loss and the demand for RBC transfusion in patients undergoing heart surgery with CPB, in a tertiary heart center of considerable operative quantity. Ulinastatin reduced the incidence of respiratory failure but did not show effect on in-hospital mortality. Moreover, we found that ulinastatin had neutral effect on long-term survival and incidence of morbidities compared with placebo and tranexamic acid. Also, the effect of reduced bleeding and transfusion by ulinastatin on long-term outcomes is evaluated for the first time.

This trial was mainly designed to evaluate the blood conservation effect of ulinastatin on heart surgery with CPB, with tranexamic acid as positive control and normal saline as negative control. Over the last 10 years, growing evidence showed that tranexamic acid effectively decreased post-operative blood loss and spared blood during this trial being conducted [20, 21, 24], tranexamic acid became the mainstay of blood conservation in cardiac surgery. Ulinastatin relieves inflammatory response induced by CPB and improves organ injuries [25–33],therefore it may provide additional advantages and become another hopeful choice for blood management. Since reports [1–3]showed that blood transfusions during or after cardiac surgery were associated with increased long-term mortality, whether the blood conservation effect of ulinastatin can be translated into the improvement of long-term outcome became intriguing. Moreover, the short- and long-term safety of ulinastatin was the major concern in this study, especially in the background of aprotinin’s withdrawal from operation room. Although there were adverse events in ulinastatin group (see Table 4), the relatively small sample size of the study limited the power to tell whether these adverse reactions are associated with ulinastatin. These account for the 10 years follow-up in the current study.

Ulinastatin is urinary trypsin inhibitor with a molecular weight of about 24,000 Da, and is extracted and purified from fresh human urine [13]. In previous studies, the PMNs degraded or inhibited the activity of fibrin, fibrinogen, platelets and coagulation factors [9–11]; ulinastatin decreased the release of elastase from PMNs and suppressed elastase activity [14]; ulinastatin also lowered the level of PMNs, tumor necrosis factor-alpha, interleukin-6 and interleukin-8 after CPB, [25] and shortened APTT and ACT in patients undergoing on-pump CABG [17]. In the current study, we demonstrated that ulinastatin decreased total post-operative blood loss by 21% and allogeneic erythrocyte transfusion requirement by 31% compared with negative control, with similar efficacy to tranexamic acid.

In the course of this trial, there were sporadic reports with negative conclusions of ulinastatin on post-operative blood loss in specific type of open heart surgery with CPB [18, 19]. Song et al. found that there were no significant improvements in coagulation profile, blood loss and transfusion requirements of patients undergoing open heart surgery with CPB by using 5000 U/Kg of ulinastatin prior to aortic cross-clamping [19], with Park et al. having similar conclusions in their study [18]. The authors inferred that, by using relative small doses of ulinastatin (5000 U/Kg), the anti-inflammatory effect of ulinastatin was overwhelmed by the inflammatory response to CPB and that CPB-induced haemodilution reduced the efficacy of ulinastatin [18, 19], therefore causing the negative results. In the present study, 1,000,000 U of ulinastatin were administrated. It was possible that increased amount of ulinastatin used in the current study suppressed the CPB-induced inflammatory response, increased the serum concentration of ulinastatin after withdraw of CPB, and therefore improved the efficacy of ulinastatin on post-operative blood conservation.

Ulinastatin has been used in open heart surgery in China and in Japan [25, 26, 28–33]to relieve the systemic inflammatory response to CPB, which was the important cause of post-operative organ dysfunctions. Bingyang et al. found that alveolo-arterial oxygen difference was significantly decreased and the duration of mechanical ventilation was shortened by ulinastatin treatment in patients undergoing CABG with CPB [25]. In a recent meta-analysis by He et al [27], in which, however, all trials included were small-sized, ulinastatin was not associated with respiratory failure, but shortened the extubation time and increased the oxygen index in patients undergoing heart surgery. In the present study, there was no significant difference in duration of mechanical ventilation. However, we observed fewer patients with post-operative respiratory failure in ulinastatin group than in other groups. Our findings added new evidence to the improvement of early post-operative pulmonary function by ulinastatin.

The study was designed to sample consecutive patients on diverse types of heart surgery with CPB but a specific surgical procedure, hoping to mimic the situation of everyday clinical practice. There was a significant shift in the composition of cardiac procedures from the beginning of this trial to nowadays, in accordance with which happened in China. For example, the percentage of valvular surgery in Fuwai Hospital has dropped from 67% in 2008 to 30% in 2017. The population in this trial was medium- or low-risked, which resulted in a much lower observed in-hospital mortality (0.47%) in accordance with the low average in-hospital mortality in this hospital, than would normally be expected with CPB alone (3.2 to 12.8%) [34]. In this situation, it was difficult to compare in-hospital and long-term mortality among groups. This is another reason why the follow-up was prolonged, from 1year originally to 10 years, to observe the time-magnified effect of different treatments on long-term survival and morbidities. However no difference was found among groups, which, on the other hand, implied that ulinastatin treatment was safe.

Cost effectiveness is an important topic in health economics and is a concern to both the health providers and the patients. Hemostatic effect of ulinastatin was rarely investigated before. As a pilot exploration, the current study focused on the efficacy and safety of the agent and, especially, with the relatively small sample size, cost effectiveness was not evaluated quantitatively in the study. This study had by far the largest sample size in studies focused on the blood conservation effect of ulinastatin. Nevertheless, the current study is medium-sized and single-centered. Further multi-centered randomized controlled clinical trials are expected to evaluate the effect of ulinastatin on blood conservation, to observe the possible adverse effects and to evaluate the cost effectiveness.

There were 14.7% patients lost in follow-up. It was a challenge to achieve a follow-up as long as 10 years. It could be attributed to the compliance of the paticipants and the large map and the unbalanced development of the country. Fuwai hospital is the National Center for Cardiovascular Disease with nationwide patients. Some participants were not well-educated and some were embarrassed in life quality, leading to an adverse adherence to the follow-up. In the past 10 years, the investigators managed to achieve a convincing rate of follow-up by face-to-face interview, email, letters, telephone call or Wechat according to different regions, educational levels and social status.

## Conclusion

In conclusion, we provided the first evidence that ulinastatin could significantly decrease the post-operative blood loss in patients undergoing heart surgery with CPB with similar efficacy to tranexamic acid in this prospective randomized controlled trial.

## Data Availability

The datasets used and/or analysed during the current study are available from the corresponding author on reasonable request.

## Abbreviations

CPBw: Cardiopulmonary bypass
PMN: Polymorphonuclear neutrophil
aPTT: Activated partial thromboplastin time.
ACT: Activated coagulation time
TXA: Tranexamic acid
PT: Prothrombin time
ICU: Intensive care unit
IABP: Intra-aortic balloon pulsation
ECMO: Extracorporeal membrane oxygenation
RBC: Red blood cell
MD: Mean difference
CI: Confidence interval
RD: Risk difference
RR: Relative risk
CABG: Coronary artery bypass graft
